# Host DNA depletion can increase the sensitivity of *Mycobacterium* spp. detection through shotgun metagenomics in sputum

**DOI:** 10.3389/fmicb.2022.949328

**Published:** 2022-10-25

**Authors:** Nienke A. Kok, Nilay Peker, Leonard Schuele, Jessica L. de Beer, John W. A. Rossen, Bhanu Sinha, Natacha Couto

**Affiliations:** ^1^University of Groningen, University Medical Center Groningen, Department of Medical Microbiology and Infection Prevention, Groningen, Netherlands; ^2^Laboratory for Medical Microbiology and Public Health, Labmicta, Hengelo, Netherlands; ^3^Laboratory of Clinical Microbiology and Infectious Diseases, Isala Hospital, Zwolle, Netherlands; ^4^Department of Pathology, University of Utah School of Medicine, Salt Lake City, UT, United States; ^5^Department of Biology and Biochemistry, The Milner Centre for Evolution, University of Bath, Bath, United Kingdom

**Keywords:** internal transcribed spacer, N-acetyl-L-cysteine, next-generation sequencing, shotgun metagenomics, saponin treatment, tuberculosis, host DNA depletion, drug-resistant

## Abstract

Identification and phenotypic drug-susceptibility testing for mycobacteria are time-consuming and challenging but essential for managing mycobacterial infections. Next-generation sequencing (NGS) technologies can increase diagnostic speed and quality, but standardization is still lacking for many aspects (e.g., unbiased extraction, host depletion, bioinformatic analysis). Targeted PCR approaches directly on sample material are limited by the number of targets that can be included. Unbiased shotgun metagenomics on direct material is hampered by the massive amount of host DNA, which should be removed to improve the microbial detection sensitivity. For this reason, we developed a method for NGS-based diagnosis of mycobacteria directly from patient material. As a model, we used the non-tuberculous mycobacterium (NTM) *Mycobacterium abscessus*. We first compared the efficiency of three different DNA extraction kits for isolating DNA (quality and concentration). The two most efficient kits were then used in a follow-up study using artificial sputum. Finally, one extraction kit was selected and further evaluated for DNA isolation from a patients’ sputum mixture spiked with *M. abscessus* at three concentrations (final concentrations 10^8^, 10^7^, 10^6^  CFU/ml). The spiked sputum samples were processed with and without saponin treatment (ST) in combination with DNAse treatment prior to bacterial DNA extraction to evaluate the recovery of bacteria and depletion of host DNA by PCR and Illumina sequencing.

While Ct values of the qPCR targeting mycobacterial ITS DNA remained rather stable, Ct values in the qPCR targeting the human β-actin gene increased by five Ct values in ST samples. In subsequent Illumina sequencing, a decrease of 89% of reads mapped to the human genome was observed in ST samples. The percentage of reads mapped to *M. abscessus* (10^8^ CFU/ml) increased by 89%, and the sequencing depth increased two times when undergoing ST.

In conclusion, the sensitivity of *M. abscessus* detection in artificial sputum was increased using a saponin pre-treatment step. The saponin followed by the DNase I treatment approach could be efficiently applied to detect and characterize mycobacterial infections, including tuberculosis, directly from sputum.

## Introduction

Mycobacteria causing infections are classified as tuberculosis-causing and non-tuberculosis mycobacteria (NTM) (Johansen et al., 2020). Tuberculosis (TB) caused by *Mycobacterium tuberculosis* is one of the leading causes of death, with over 1 million deaths worldwide yearly (World Health Organization, 2019). Although the *M. tuberculosis* mutation rate is relatively low under optimized treatment conditions, multidrug-resistant (MDR) and extensively drug-resistant (XDR) *M. tuberculosis* are advancing rapidly (World Health Organization, 2018). A prompt and accurate characterization of the complete resistance profile of TB, which currently takes 2–6 weeks or longer in routine diagnostic laboratories, is critical to optimize appropriate antimicrobial therapy, thereby reducing antimicrobial selection pressure (World Health Organization, 2021). To implement a faster method to detect and characterize TB and NTM, broad genotypic testing of direct material using molecular techniques such as polymerase chain reaction (PCR) is already applied in many laboratories. These sensitive molecular techniques can provide results within hours and have been successfully implemented even in low resource settings (World Health Organization, 2018). Next-generation sequencing (NGS), on the other hand, is being used for comprehensive genotypic drug susceptibility testing (DST) and is usually performed on culture isolates as direct testing of clinical samples by shotgun metagenomics (SMg) results in the sequencing of vast amounts of human DNA (World Health Organization, 2021). This hampers the sensitivity of detection and the genome coverage necessary for predicting genotypic drug susceptibility and inferring genetic relatedness.

Enrichment of bacterial DNA by human DNA depletion has improved pathogen detection by NGS (Hasan et al., 2016). In this study, human DNA was depleted using a saponin pre-treatment (ST) step. Saponin is a secondary plant compound that permeabilizes cell membranes by targeting cholesterol. The formation of a saponin-cholesterol complex in the cell membrane causes pores in the cell wall of eukaryotic cells resulting in membrane instability (Böttger and Melzig, 2013). Because bacteria do not have cholesterol, they are not affected by this treatment. The ST protocol used in this study is based on a recently published ST protocol optimized for nanopore sequencing of lower respiratory infections, but that did not include mycobacterial species (Charalampous et al., 2019). Since saponin has been reported to have a negative effect on the DNA recovery of some bacterial species (Street et al., 2020), we wanted to test the saponin protocol in mycobacteria-containing samples. Additionally, the efficiency of three different DNA extraction kits was studied. *Mycobacterium abscessus* is a rapidly growing mycobacterium associated with pulmonary infections (Brown-Elliott and Philley, 2017) that does not need to be processed under Biosafety Level-3 (BSL 3) laboratory conditions. Additionally, *M. abscessus* has been shown to share conserved strategies for host adaptation and persistence with *M. tuberculosis* (Low et al., 2017). Therefore, we developed an NGS-based method for detecting the presence and resistance patterns of *M. abscessus* (ATCC 19977) as a model for mycobacteria. Overall, we aimed to develop an optimized SMg protocol that can be applied to detect and characterize mycobacterial infections.

## Materials and methods

### *Mycobacterium abscessus* isolate

*Mycobacterium abscessus* ATCC 19977 (ATCC, London, United Kingdom) was initially grown on blood agar plates (product and manufacturer) at 37°C for 3–5 days until bacterial growth was visible. A stock suspension of *M. abscessus* (1.21 × 10^9^ CFU/ml) was prepared in 1 × PBS (Thermo Scientific, Waltham, MA, United States), and 10-fold serial dilutions were prepared in duplicate, ranging from a concentration of 1.21 × 10^7^ to 1.21 × 10^9^ CFU/ml. Bacterial concentrations were confirmed by plating eight serial dilutions on blood agar plates (BD, Franklin Lakes, United States; 100 μl/plate) and counting plates that showed 30–300 colonies.

### Comparison of three different extraction kits using mycobacterial suspensions

DNA extraction from *M. abscessus* isolates was performed using the (i) DNeasy UltraClean Microbial kit (Qiagen Benelux, Venlo, Netherlands; DNeasy), (ii) QIAamp DNA Mini kit (Qiagen; DNA Mini), and (iii) AllPrep PowerFecal DNA/RNA kit (Qiagen; AllPrep). All kits were used following the respective manufacturer’s recommended protocol with the following minor modifications (Qiagen, 2016, 2017, 2018a): for all kits, the starting volume was 200 μl, and the DNA was eluted twice to a total elution volume of 30 μl of the respective elution buffer provided by the kits; the AllPrep protocol was adjusted using 2 ml PowerBead Pro tubes (cat. No. 19301; Qiagen) instead of the provided Microbial Lysis Tubes; centrifugation steps were adjusted to a maximum of 10,000 ×*g*, and the centrifugation time was doubled if a higher centrifugation force was required (Merck KGaA, 2018). The purity of DNA was analyzed using the NanoDrop^™^ 2000 spectrophotometer (Thermo Scientific), and DNA concentrations were measured using the Qubit^™^ 2.0 and the dsDNA High Sensitivity Assay Kit (Thermo Scientific). Afterwards, a quantitative PCR (qPCR) targeting the 16S rRNA gene was performed using the Applied Biosystems 7500 Real-Time PCR System (Thermo Scientific) with the primers listed in Supplementary Table S1.

### Spiking experiments

#### *Mycobacterium abscessus* spiked in artificial sputum

Spike-in experiments were initially conducted using artificial sputum, consisting of mucin, salmon sperm, egg yolk and salts (the protocol is available at: https://protocols.scienceexchange.com/protocols/artificial-sputum-medium) (Sriramulu et al., 2005). After preparation, the artificial sputum was aliquoted and stored at –80°C. Artificial sputum samples (250 μl) were spiked in triplicate with 50 μl of the *M. abscessus* bacterial stock suspension at two initial dilutions (1.21 × 10^9^ and 1.21 × 10^8^ CFU/ml; final concentrations of 2.02 × 10^8^ and 2.02 × 10^7^ CFU/ml, respectively). Prior to DNA extraction, 300 μl of spiked samples were treated with 200 μl of a 2.5% saponin solution (Merck; diluted in 1 × PBS and sterile filtered). Samples were vortexed thoroughly and incubated at room temperature for 10 min to promote host cell lysis. Subsequently, 350 μl of nuclease-free water (Merck) was added, and samples were incubated for 30 s. For the osmotic shock, 12 μl of 5 M NaCl (Merck) were added to all samples. Samples were centrifuged at 6,000 × *g* for 5 min, and pellets were resuspended in 100 μl 1 × PBS (Thermo Scientific). Next, 15 μl DNase I [20,000 Units (50 to 375 U/μl); Thermo Scientific] and 11 μl of 10 × DNase buffer (Thermo Scientific) were added to digest free DNA. Samples were incubated in a thermomixer (Eppendorf, Hamburg, Germany) with shaking at 800 rpm at 37°C for 30 min. After incubation, samples were centrifuged at 6,000 × *g* for 3 min, and pellets were washed twice with 800 μl 1 × PBS (Sriramulu et al., 2005). Finally, pellets were resuspended in the lysis buffer of the AllPrep and DNeasy kits, following the respective manufacturer’s instructions. Extracted DNA was eluted twice with 15 μl of elution buffer and centrifuged at 6,000 × *g* for 1 min. Concentration and purity of DNA were measured using the Qubit^™^ 2.0 with the dsDNA High Sensitivity Assay Kit (Thermo Scientific) and the NanoDrop^™^ 2000 spectrophotometer (Thermo Scientific), respectively. Fragment length of ST and saponin not-treated (NT) samples spiked with 1.21 × 10^9^ CFU/ml *M. abscessus* was measured using the Agilent 2,200 TapeStation (Agilent, Santa Clara, CA, United States). Then, qPCR targeting the human β-actin gene and 16S rRNA gene (Supplementary Table S1) was performed using the LightCycler 480 Instrument (Roche Diagnostics).

#### *Mycobacterium abscessus* spiked in human sputum

**Figure 1:**
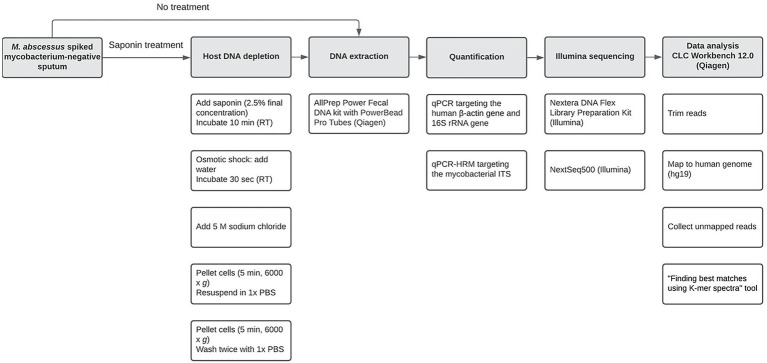
Workflow used in the current study from spiking *Mycobacterium*-negative sputum until data analysis. Open boxes in a column represent specific subsequent sub-steps (from top to bottom) for major steps (shaded boxes).

The workflow is illustrated in Figure 1. *Mycobacterium*-negative sputum samples screened for using an internal transcribed spacer high resolution melting curve (ITS-HRM) PCR, using a mycobacterial specific primer set (Akkerman et al., 2013) from different patients analyzed for general microbiological diagnostics in Labmicta (Hengelo, Netherlands) were pooled until a total sputum volume of approximately 15 ml was obtained. To promote mucolysis, approximately 1 gram, depending on the viscosity of the sputum, of N-Acetyl-L-Cysteine (NALC; Merck, Darmstadt, Germany) was added and vortexed until liquefied. Samples were processed as described above (50 μl of bacterial stock was spiked into 250 μl sputum), an additional dilution was used (1.21 × 10^7^ CFU/ml), and experiments were performed in duplicate. Only the AllPrep kit was tested further using this protocol based on prior validation experiments. Additionally, the ITS-HRM PCR was performed using a primer/probe set (Supplementary Table S1) that specifically detects mycobacterial DNA using the Applied Biosystems 7,500 Real-Time PCR System (Thermo Scientific).

#### Next-generation sequencing of *Mycobacterium abscessus*-spiked sputum samples

DNA pellets of duplicate samples were pooled and purified with 1.8 volumes of AMPure XP Beads (Beckman Coulter, Indianapolis, United States) according to the manufacturer’s instructions and eluted in 20 μl water (Coulter, 2016). Sequencing libraries were prepared using the Nextera DNA Flex Library Prep Kit according to the manufacturer’s protocol (Illumina, San Diego, CA, United States) (Illumina, 2019) and sequenced using the Illumina NextSeq 500 (Illumina) to generate 2 × 150 bp paired-end reads. The *M. abscessus* average sequencing depth was 40.4 (maximum 85.5 and minimum 3.4; Supplementary Table S2).

#### Bioinformatics

Sequencing reads (fastq files) were analyzed using the CLC Genomics Workbench v21.0.5 (Qiagen) (Qiagen, 2018b) with default settings and parameters unless mentioned otherwise. First, the paired-end reads were quality and adapter trimmed. Then they were mapped (Map Reads to Reference v1.8) to the reference human genome (hg19), and unmapped reads were analyzed using the “Find Best Matches using K-mer Spectra” tool v1.4 (CLC Microbial Genomics Module 21.1) and an in-house microbial database (2018-10-18) build-in CLC Genomics Workbench. The relative abundance of *M. abscessus* was compared between ST and NT sputum spiked samples. For microbial characterization, reads were also uploaded to the One Codex Platform (One Codex, San Francisco, CA, United States; Minot et al., 2015).

### Statistical analysis

Statistical analysis was performed using Prism 9 for macOS v9.3.0 (GraphPad Software, San Diego, CA, United States). Results were considered significant at *p* < 0.05. For numerical variables, the parametric paired t-test (following Shapiro–Wilk normality test) or nonparametric Mann–Whitney or Wilcoxon signed rank tests were used to compare groups.

### Medical ethical approval

Sputum samples not used for routine diagnostics were collected with approval from the Medical Ethics Review Board of UMCG (METc number 2018/393). The study was performed in adherence to the guidelines of the Declaration of Helsinki and local regulations, and all patient data were treated pseudo-anonymously.

## Results and discussion

### Selecting the most effective DNA extraction kits

Bias is a pervasive problem when performing DNA extraction (McCarthy et al., 2015). An important factor is the difference in lysis efficiencies of different bacterial populations, which vary depending on the extraction protocol (McCarthy et al., 2015). Therefore, we tested three different extraction kits on *M. abscessus* suspensions. The quality and concentration of DNA using the three different extraction kits were compared. DNA concentrations were highest when using the DNeasy and AllPrep kits (Table 1).

**Table 1:** Quantification and quality of DNA extracted through three commercially available kits (DNeasy, DNA Mini, and AllPrep).

Concentration *M. abscessus* (CFU/ml)	DNeasy			DNA Mini			AllPrep		
	ng/μl	260/280	260/230	ng/μl	260/280	260/230	ng/μl	260/280	260/230
1.21 × 10^9^	> 60.00	1.89	2.26	1.17	1.36	0.75	48.30	1.53	1.51
	> 60.00	1.83	2.03	0.90	2.48	0.35	40.00	1.97	1.35
1.21 × 10^8^	6.79	1.74	0.97	0.39	2.60	0.16	6.66	1.83	0.29
	11.80	1.79	1.31	0.41	0.74	0.65	7.88	2.77	0.23
1.21 × 10^7^	0.29	1.29	0.25	0.10	3.51	0.12	0.47	2.69	0.10
	0.38	3.96	0.32	0.12	5.24	0.12	0.52	0.71	0.12

Rows per concentration represent replicates.

**Figure 2:**
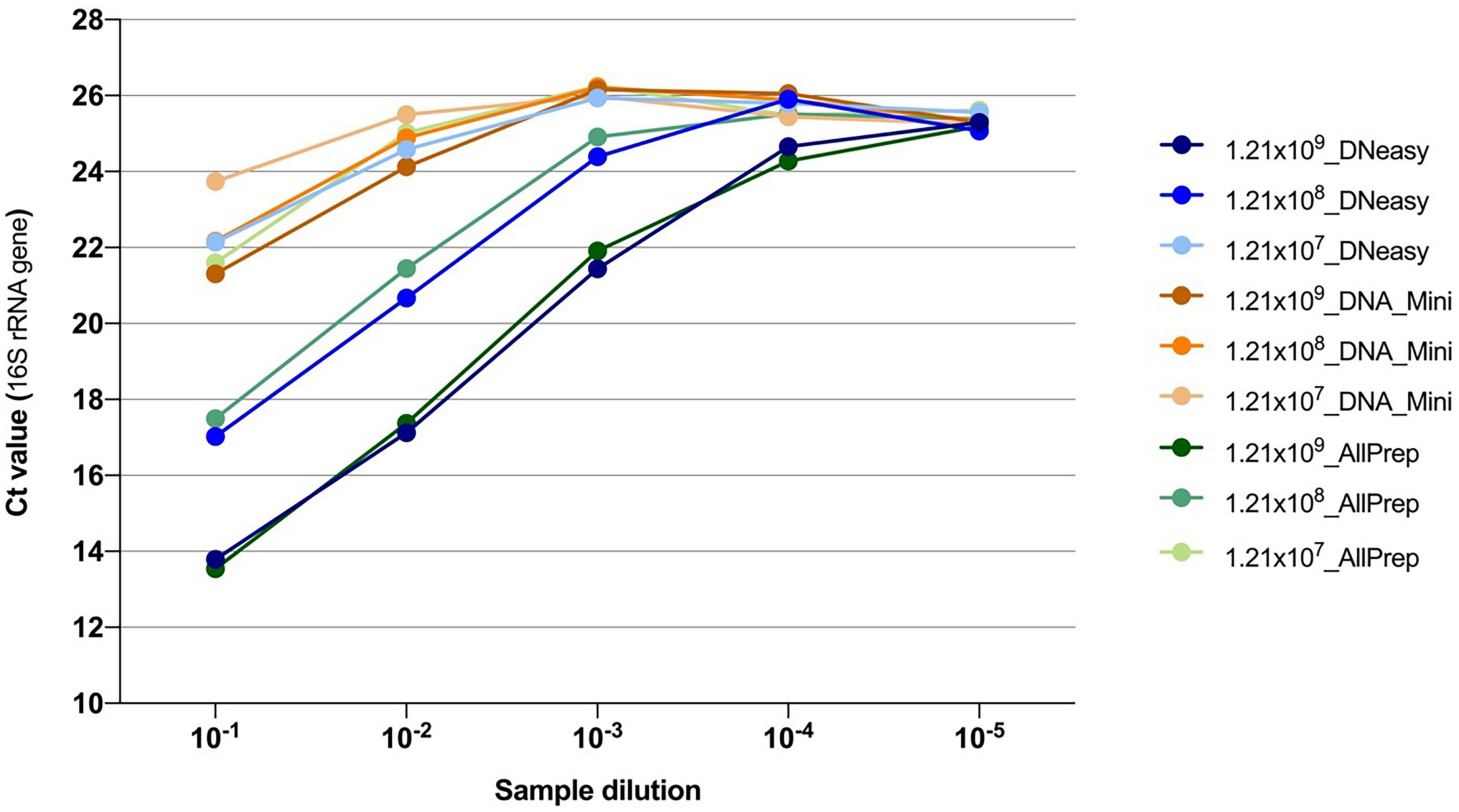
Quantification of the 16S rRNA gene using qPCR from DNA extractions. Colors indicate the combination of the concentration of the bacteria and the kit used for extracting the DNA. After extraction, DNA samples were 10-fold serially diluted, and five dilutions were subsequently tested.

Additionally, quantification of the 16S rRNA gene by qPCR showed that the DNeasy and AllPrep kits both yielded more bacterial DNA than the Mini kit (Figure 2).

Interestingly, in a recent study comparing DNA extraction strategies for livestock fecal microbiota, the Allprep PowerFecal DNA/RNA Kit (Qiagen) yielded the lowest concentrations of DNA and the lowest quality (260/280 values were all outside the acceptable range of 1.8–2.0) (Wegl et al., 2021). This is discordant with the results obtained in the present study. However, it should be noted that the sample matrix and complexity can differ substantially between studies. Moreover, we substituted the Microbial Lysis Tubes in the Allprep kit with the PowerBead Pro tubes to increase DNA extraction efficiency, improving DNA concentration and quality. Since the DNeasy and AllPrep kits showed the most promising DNA yield and quality results, they were used in the follow-up study using artificial sputum.

### Effect of saponin treatment on recovery of *Mycobacterium abscessus* DNA

Host depletion or target enrichment is an important step in a metagenomics workflow to increase the sensitivity of the method (Schuele et al., 2021). Saponin treatment effectively depletes host DNA in respiratory samples with a maximum of 10^4^-fold depletion (median 600-fold) of human DNA (Charalampous et al., 2019). However, saponin has been reported to have a negative impact on the DNA recovery of certain bacterial species (Street et al., 2020). In this study, we opted for a host depletion step because the method developed here could potentially be applied for the detection and characterization of multiple respiratory pathogens (not only mycobacteria) and even for microbiome studies.

**Figure 3:**
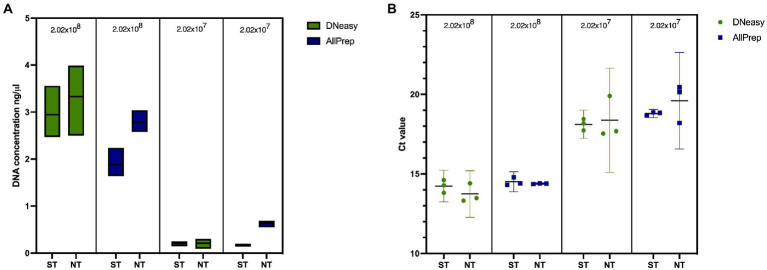
Effect of saponin and two DNA extraction kits on artificial sputum samples in terms of **(A)** DNA concentration measured with Qubit^TM^ 2.0 and **(B)** qPCR targeting the 16S rRNA gene in spiked artificial sputum. ST, saponin treated; NT, not treated.

We first tested our saponin protocol using artificial sputum to understand the effect of saponin treatment on yields of mycobacterial DNA. As shown in Figure 3A and Supplementary Table S3, ST resulted in slightly lower DNA concentrations, independent of the extraction kit used, although differences were not statistically significant (*p* > 0.05). Additionally, this did not result in significantly (*p* > 0.05) different 16S rRNA gene quantifications (Figure 3B; Supplementary Table S3), suggesting that although higher DNA concentrations were obtained in NT samples, this was not reflected in higher mycobacterial DNA concentrations and probably reflects the extraction of salmon DNA, which is digested in the ST method by the addition of DNase I. When comparing ST and NT samples, the fragment lengths of extracted DNA were similar (*μ* = 15,567 bp compared to *μ* = 15,945 bp, respectively; Supplementary Figure S1).

Lower DNA concentrations were obtained when extracting samples with the AllPrep kit than with the DNeasy kit. However, these differences were less prominent after quantifying the *16S rRNA* gene (Figure 3B; Supplementary Table S3). Fragment lengths were longer when using the AllPrep kit (*μ* = 17,603 bp) than with the DNeasy kit (*μ* = 13,912 bp), although this difference was not statistically significant (*p* = 0.3333; Supplementary Figure S1). Contrary to the DNeasy kit, the AllPrep kit allows for differential nucleic acid extraction of DNA and RNA in separate eluates, which allows the detection of RNA viruses or look at indicators of host response (if no depletion step is used). The results provided by this kit were more repeatable (with less variation between replicates). Therefore, we decided to use the AllPrep kit for experiments with human sputum.

### Effect of saponin treatment on human DNA depletion

**Figure 4:**
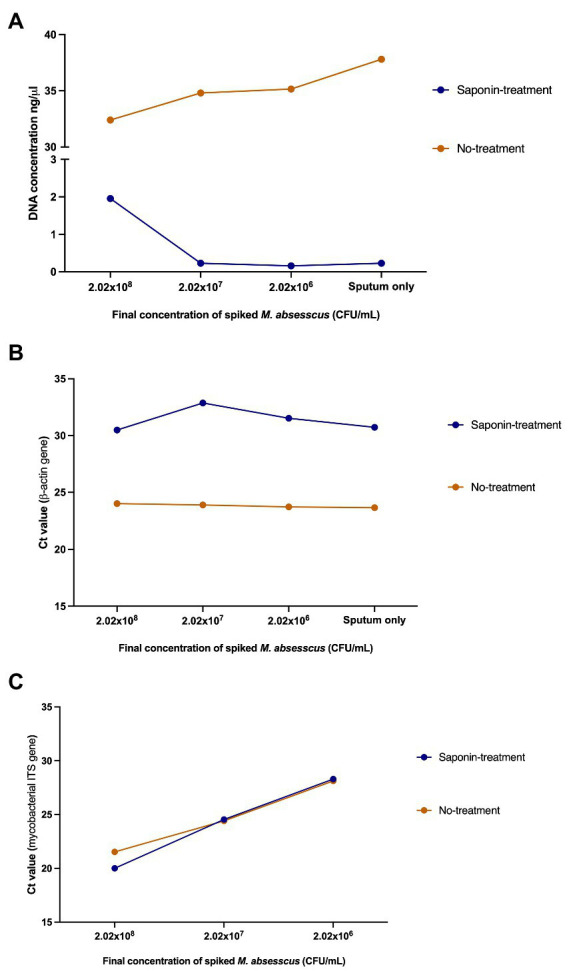
Effect of saponin treatment on human and *M. abscessus* DNA. **(A)** DNA quantification using Qubit^TM^ v2.0, **(B)** qPCR targeting the human β-actin gene, and **(C)** qPCR targeting the mycobacterial internal transcribed spacer (ITS) on spiked human sputum (sputum only: non-spiked sputum sample). Concentrations and Ct values shown in this figure represent the average of the duplicates.

After evaluating the effect of saponin treatment on the recovery of *M. abscessus* DNA, we tested the effect of saponin treatment on the differential lysis of human cells and subsequently depletion of human DNA. To comply with ethical standards and for practical reasons, we pooled mycobacterial-negative sputum samples from different individuals. Higher DNA concentrations were obtained in the NT samples (Figure 4A). The human β-actin gene was detectable five cycles earlier in the NT conditions indicating a higher amount of human DNA in these samples (Figure 4B). This difference was statistically significant (*p* = 0.0008; paired *t*-test) and suggested that saponin had a significant lytic effect on human cells, which then resulted in lysis of the human DNA by the DNase I treatment. The effect of the ST on *M. abscessus* was negligible looking at the Ct values of the qPCR targeting the mycobacterial ITS, which hardly changed in ST compared to NT samples (Figure 4C), indicating it had no negative impact on *M. abscessus* DNA recovery.

**Figure 5:**
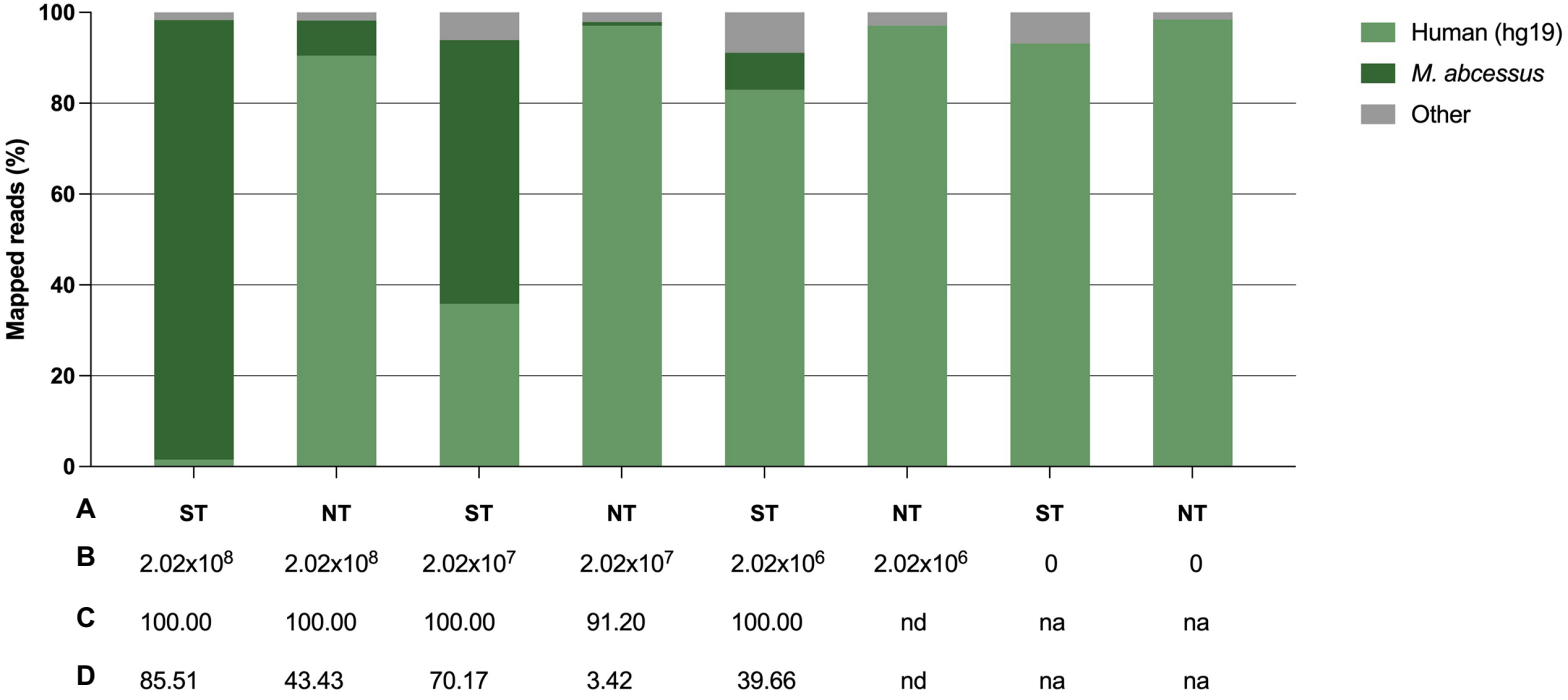
Effect of saponin treatment (depleting host DNA) on sputum spiked with *M. abscessus* at three different concentrations. **(A)** Condition, i.e., saponin treated (ST) or non-treated (NT), **(B)** Final concentration of spiked *M. abscessus* (CFU/ml), **(C)** *M. abscessus* genome coverage (%), **(D)** *M. abscessus* mean sequencing depth (average number of reads per sites). 0 concentration refers to non-spiked sputum samples. na, not applicable; nd, not detected.

To confirm the previously obtained qPCR results, we sequenced the samples and assessed the efficiency of saponin in depleting host DNA by comparing the proportion of *M. abscessus* and human reads between ST and NT samples. In ST samples with a final *M. abscessus* concentration of 2.02 × 10^8^ CFU/ml, 2.02 × 10^7^ CFU/ml and 2.02 × 10^6^ CFU/ml, approximately 1.5, 35.8 and 82.9% of the reads mapped to the human genome, respectively, compared to 90.5, 97.1 and 97.1% in the NT samples, respectively (Figure 5; Supplementary Table S2).

A difference in the proportion of reads mapped to the human genome, although smaller, was also noted in treated and non-treated non-spiked control samples (sputum only). Overall, there was a 60-fold, 2.7-fold and 1.2-fold decrease in the proportion of human reads in ST samples with a final *M. abscessus* concentration of 2.02 × 10^8^ CFU/ml, 2.02 × 10^7^ CFU/ml and 2.02 × 10^6^ CFU/ml, respectively. There was a 12.5-fold, 74.5-fold increase in the proportion of *M. abscessus* reads in ST samples with a final *M. abscessus* concentration of 2.02 × 10^8^ CFU/ml and 2.02 × 10^7^ CFU/ml, respectively. In samples with a final *M. abscessus* concentration of 2.02 × 10^6^ CFU/ml, 8.1% of the reads were classified for *M. abscessus* in ST samples, whereas *M. abscessus* could not be detected in NT samples. At lower concentrations of *M. abscessus* (2.02 × 10^7^ and 2.02 × 10^6^ CFU/ml), full genome coverage was only obtained after ST. ST also resulted in a higher sequencing depth for *M. abscessus*. High genome coverage is essential to identify bacteria to the species level, gene sequences associated with antimicrobial resistance, and for accurate variant (or SNP) calling for outbreak management.

Other methods of human DNA depletion have been tested in *M. tuberculosis-*positive samples. One was based on osmotic lysis by adding a large volume of sterile water to a sputum sample to increase osmotic pressure and cause human cells to burst while leaving the more robust *M. tuberculosis* cells intact (Doughty et al., 2014). A DNase enzyme is then used to degrade the released human DNA prior to *M. tuberculosis* DNA extraction. However, this approach is severely limited for acquiring sufficient (due to the high host to bacteria cell ratio) bacterial DNA from low load samples without enrichment (Doughty et al., 2014) and some co-infection causing pathogens of interest, such as Gram-negative bacteria, are also susceptible to osmotic pressure (Bachmann et al., 2018). Votintseva et al. (2017) used a commercial kit that selectively lyses human and other eukaryotic cells in *M. tuberculosis*-positive respiratory samples. This kit is based on a chaotropic buffer that selectively lyses eukaryotic cells while keeping the bacterial cells intact, however, the identity of the chaotropic buffer is not disclosed by the manufacturer (Bachmann et al., 2018). They were able to identify *M. tuberculosis* in all samples from which DNA was successfully extracted, and sufficient data for antimicrobial susceptibility prediction was obtained from 62% of the samples (Votintseva et al., 2017). The sample composition affected the number of *M. tuberculosis* reads and depended on the amount of human and bacterial DNA in the sample (Votintseva et al., 2017). Although, the use of certain commercial extraction kits can reduce the turnaround time and are easier to standardize and validate (Votintseva et al., 2017), they are usually more costly and might not be suitable for low- and middle-income settings where there is unavailability or inaccessibility of certain kits/reagents (Boro and Stoll, 2022).

### Effect of saponin treatment on recovery of DNA of other microbes

**Figure 6:**
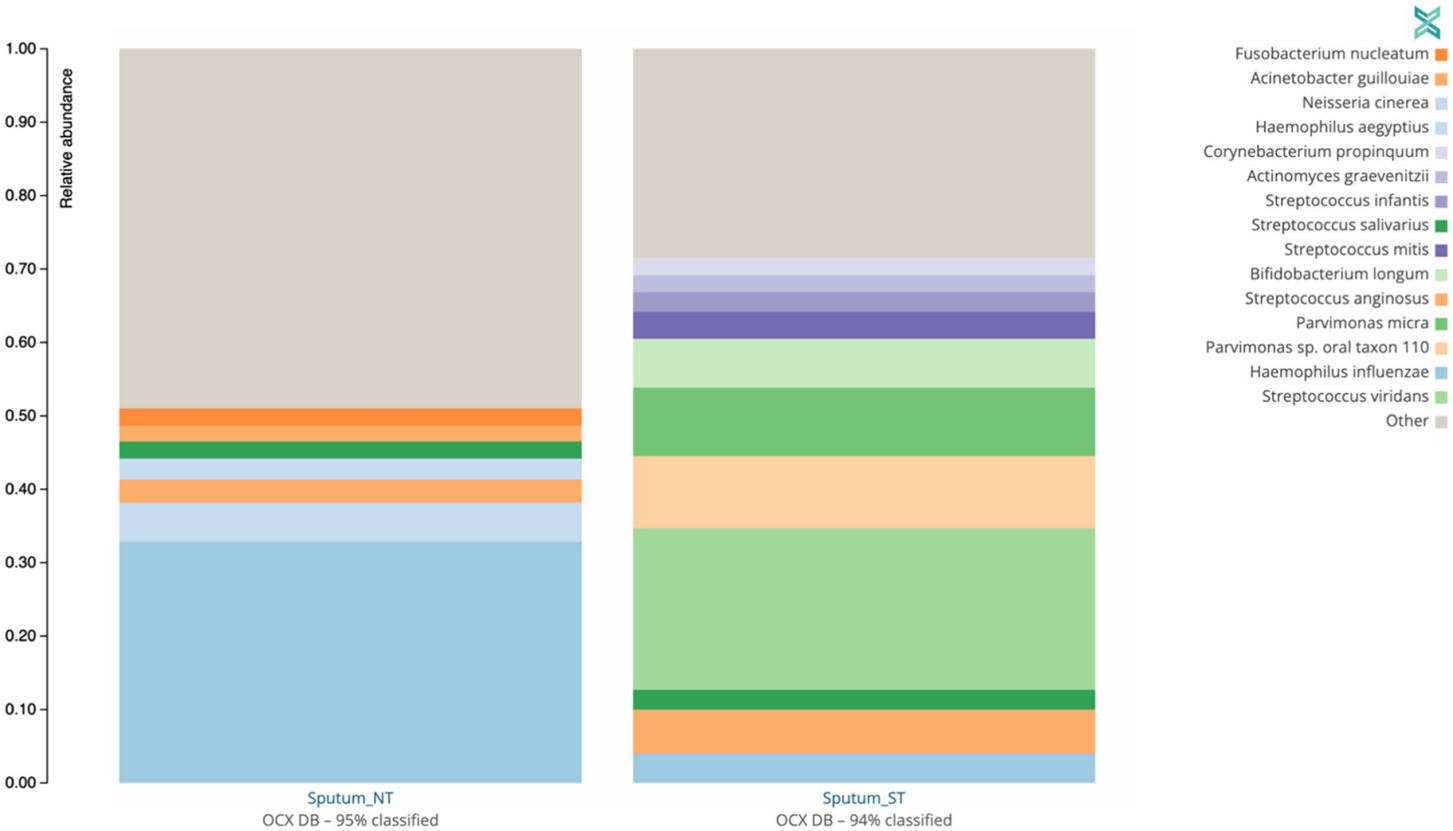

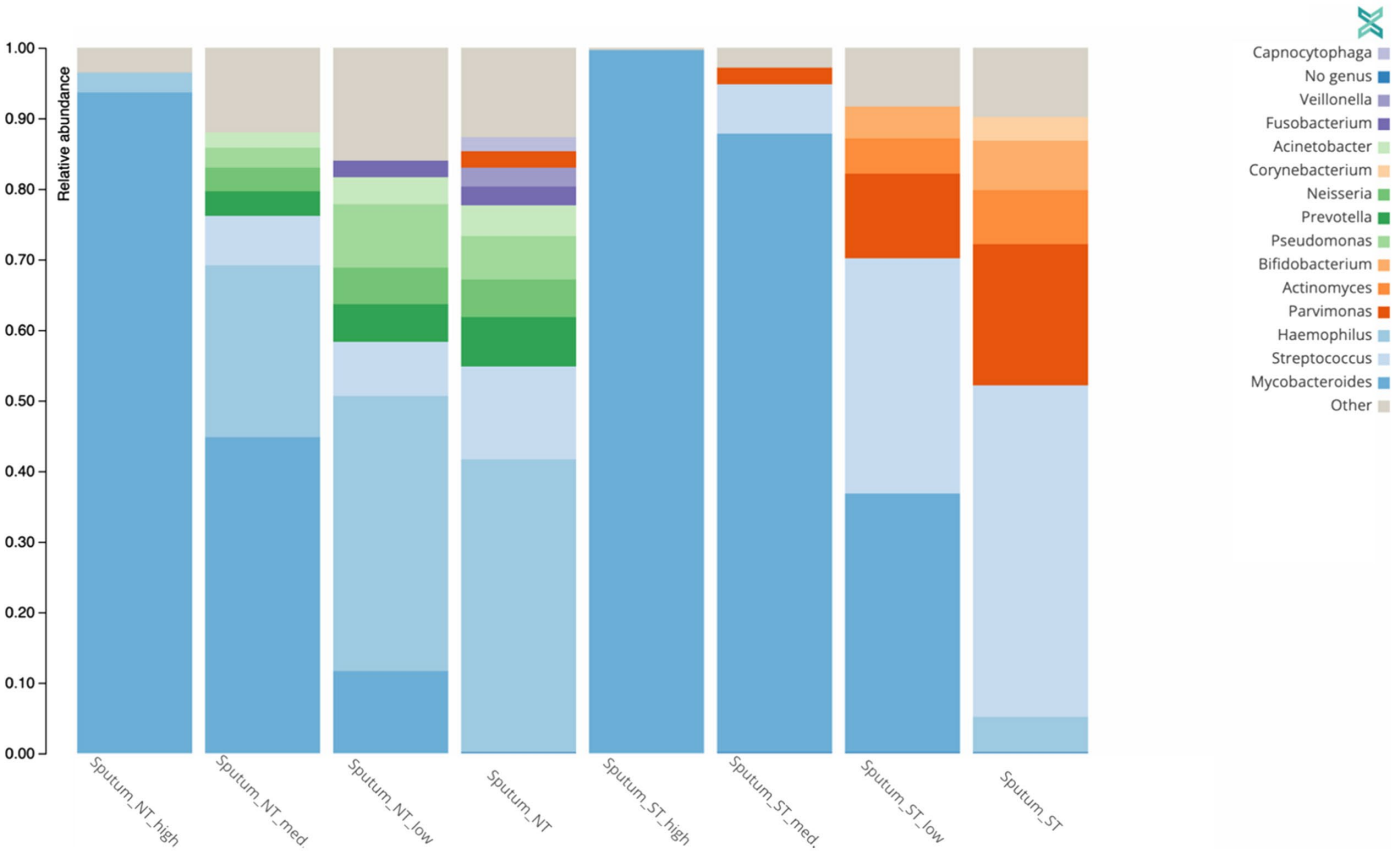

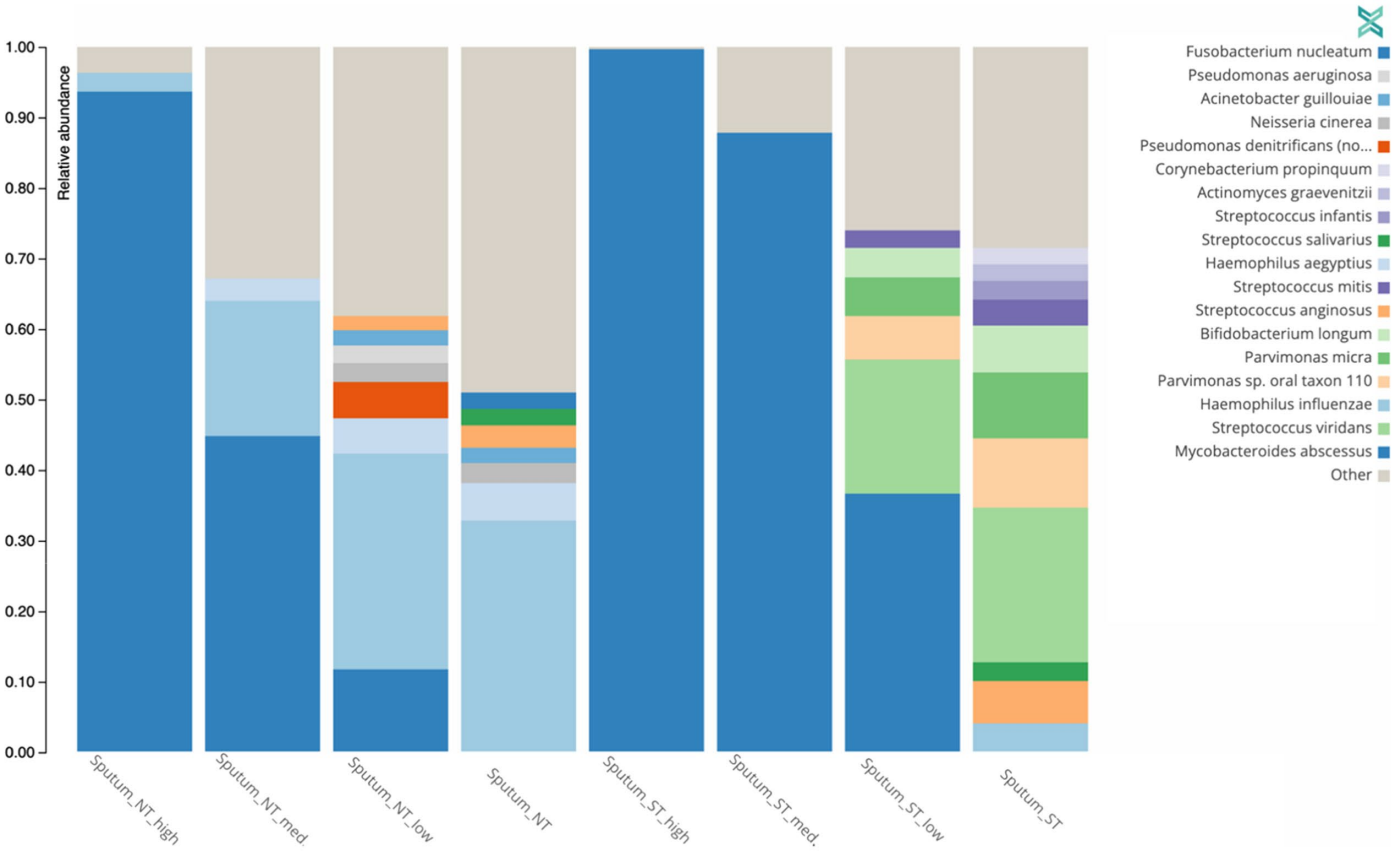
Effect of saponin treatment (ST) on microbiota present in patient sputa. Non-spiked sputa **(A)** and sputa spiked **(B,C)** with different concentrations of *M. abscessus* (high: 2.02  ×  10^8^  CFU/ml, med: 2.02  × 10^7^ CFU/ml, low: 2.02  ×  10^6^ CFU/ml). Subsequently, sputa were incubated (ST) or not (NT) with saponin followed by DNAse treatment. Relative abundance of bacterial genera **(A,B)** or species **(C)** with at least 2% reads are shown. Other refers to low abundant species.

To understand the broader impact of human DNA depletion, we evaluated the effect of saponin treatment followed by DNase I digestion on other microbial species in the sputum samples. Using the One Codex platform (Minot et al., 2015), we determined the presence of other bacterial species (Figure 6) and viruses (Supplementary Figure S3) in the sputum samples used for the spiking experiments.

The two microorganisms predominantly found in non-spiked ST and NT sputa were *Streptococcus viridans* (22%) and *Haemophilus influenzae* (33%), respectively (Figure 6A). Similarly, *S. viridians* and *H. influenzae* were mostly found in *M. abscessus* spiked ST and NT sputum, respectively (Figures 6B,C). These results suggest that saponin followed by DNase I treatment depletes Gram-negative bacteria. Saponin treatment combined with a DNase I step is an efficient option to deplete host DNA (Bachmann et al., 2018; Charalampous et al., 2019; Boro and Stoll, 2022), however, the effect on different bacterial species varies depending on the study. In a study optimizing culture-independent genome sequencing, saponin treatment resulted in almost undetectable levels of human DNA with minimal loss of *Bordetella pertussis* DNA (Fong et al., 2020). In another study, however, depletion of human DNA with saponin treatment increased *Neisseria gonorrhoeae* yields in simulated infections but decreased yields in clinical samples (Osei-Wusu et al., 2021). Both species are Gram-negative bacteria. Hence, variations in the effect of saponin treatment cannot be solely explained by differences in the cell wall structure.

Furthermore, another study examining the effect of saponin followed by DNase I treatment also showed an increase in the proportion of reads detected for DNA viruses (Hasan et al., 2016). Although we did not mainly focus on viruses in our study, we also observed an increase in the proportion of reads detected for viruses in ST samples (Supplementary Figure S3). However, our protocol also included osmotic lysis and centrifugation, which might have degraded viruses susceptible to osmotic stress. A balance between effective host depletion and minimum negative impact on microbial cells (and therefore microbial DNA) must be achieved while designing a saponin-DNase I treatment protocol. Our study shows that the protocol used here did not affect the overall mycobacterial DNA extraction, while significantly reducing the human DNA load, which resulted in higher mycobacterial read counts and higher sensitivity. Our protocol, nonetheless, might not be applicable to other bacterial species such as *H. influenzae.*

A limitation of this study is the restricted number of bacterial dilutions of *M. abscessus* tested. Mycobacteria can be present in clinical samples at low concentrations (10^1^–10^3^ CFU/ml) (Walker et al., 2015), which is substantially lower than the dilutions tested using this protocol. However, in this study, we aimed to quantify the effect of saponin treatment on human cells and *M. abscessus*, which would have been challenging in samples with unquantifiable DNA concentrations. It is worth to mention, however, that in the study conducted by Charalampous et al. (2019) using a similar extraction method, the limit of detection (LoD) of the method ranged from 10^3^ to 10^5^ CFU/ml for *E. coli* and *S. aureus* and depended on different levels of background commensal/human DNA. Accordingly, and in another study using SMg, researchers were able to identify and characterize *M. tuberculosis* in samples with low AFB scores (+1; approx. 5,000 bacilli per milliliter of specimen) and no growth in MGIT culture (Votintseva et al., 2017). Taking this into consideration and assuming that similar LoDs would be identified for mycobacteria, the LoD is probably the main drawback of our current SMg protocol. However, we believe the method proposed here is of substantial diagnostic/therapeutic value because it has the potential to complement more sensitive diagnostic tests (if there is a strong suspicion of TB or NTM infection even though culture/microscopy is negative) and speed up the turnaround time for DST and/or phylogenetic inference in case of a suspected resistant strain that would lead to therapeutic failure (e.g., recurrent mycobacterial infection) or in case an outbreak is suspected, respectively.

The second limitation of this study was the lack of clinical *Mycobacterium*-positive sputum samples to test our protocol. We attempted to overcome this limitation by spiking sputum from patients with mycobacteria, but we acknowledge that this may not entirely reflect the clinical situation.

## Conclusion

The present study aimed to develop an optimized extraction protocol to complement available TB/NTM-diagnostic tests for faster susceptibility testing and outbreak management of mycobacterial infections using NGS. We showed that saponin combined with DNase I treatment was an effective method for host depletion, by decreasing the availability of human DNA for PCR amplification during library preparation, and with minimal impact on the recovery of mycobacterial DNA as opposed to previous observations for other pathogens. SMg can complement other diagnostic tests (e.g., culture, microscopy, POC tests, PCR), by decreasing the turnaround time for a comprehensive drug susceptibility testing and by combining detailed and comprehensive diagnostic information (Peker et al., 2021), including typing of isolates, in a single test. It has the potential to provide increased sensitivity, potential for automation, and further decreased time to result in the future.

## Data availability statement

The datasets presented in this study can be found in online repositories. The names of the repository/repositories and accession number(s) can be found at: https://www.ncbi.nlm.nih.gov/, PRJNA823663.

## Author contributions

NC, JR, BS, and NP conceived and supervised the study. NK, NP, and LS adjusted the host depletion pre-treatment protocol and performed the sequencing experiments. NK, NP, and JB performed the host depletion and DNA extraction. NK, NP, LS, and NC analyzed the data. JB contributed with sputum samples. NK, NP, LS, and NC wrote the draft manuscript. All authors contributed to the article and approved the submitted version.

## Funding

This study was funded in part by the European Union’s Horizon 2020 Research and Innovation Program under the Marie Skłodowska-Curie grant agreement 713660 (MSCA-COFUND-2015-DP “Pronkjewail”) and by the Stichting Beatrixoord Noord-Nederland.

## Conflict of interest

NK is currently employed by Becton, Dickinson and Company. JR and NC consulted for IDbyDNA. JR consults for ARES-Genetics. NC consults for the University of Oxford. However, this did not influence the writing of the manuscript.

The remaining authors declare that the research was conducted in the absence of any commercial or financial relationships that could be construed as a potential conflict of interest.

## Publisher’s note

All claims expressed in this article are solely those of the authors and do not necessarily represent those of their affiliated organizations, or those of the publisher, the editors and the reviewers. Any product that may be evaluated in this article, or claim that may be made by its manufacturer, is not guaranteed or endorsed by the publisher.

## Abbreviations

ITS: internal transcribed spacer
HRM: high resolution melting curve
MDR: multidrug-resistant
NALC: N-acetyl-L-cysteine
NGS: next-generation sequencing
NT: not-treated
NTM: non-tuberculous mycobacteria
qPCR: quantitative PCR
SMg: Shotgun metagenomics
ST: saponin treated
TB: tuberculosis
WHO: World Health Organization
XDR: extensively-drug resistant
