# Supplementary material for: Host DNA depletion can increase the sensitivity of Mycobacterium spp. detection through shotgun metagenomics in sputum
Source: Front Microbiol. 2022 Oct 25;13:949328. doi: 10.3389/fmicb.2022.949328 (PMC9642804; doi:10.3389/fmicb.2022.949328)

**Supplementary Data**

**Table S1.** Primers used for qPCR.

| **Target** | **Type** | **Sequence** |
| --- | --- | --- |
| Bacterial 16S rRNA gene | Forward | GACTACHVGGGTATCTAATCC |
| Bacterial 16S rRNA gene | Reverse | CCT-ACG-GGN-GGCWGCAG |
| Human β-actin gene | Forward | GCT-GCT-TCT-CAT-TGT-CTC |
| Human β-actin gene | Reverse | GCC-AGG-AGA-ATG-AGG-TGG-TC |
| Mycobacteria (ITS-HRM)  (Tib-Molbiol) | Forward | ACC-TCC-TTT-CTA-AGG-AGC-ACC |
| Mycobacteria (ITS-HRM)  (Tib-Molbiol) | Reverse | GAT-GCT-CGC-AAC-CAC-TAT-CCA |

**Table S2.** Summary of sequencing reads.

|  | **Trimming report** | | | | **Mapping against hg19** | | **Finding best matches using k-mer spectra** | | |
| --- | --- | --- | --- | --- | --- | --- | --- | --- | --- |
| Sample ID | Number of reads before trimming | Average read length before trimming | Number of reads after trimming | Average read length after trimming | Number of mapped reads | Proportion of mapped reads (%) | Proportion of *M. abscessus* reads (%) | Average coverage | Proportion of reference covered (%) |
| ST_10^9^ CFU/mL | 3,080,976 | 145.68 | 3,074,173 | 145.78 | 46,438 | 1.51 | 96.82 | 85.51 | 100 |
| NT_10^9^ CFU/mL | 24,563,900 | 124.17 | 24,195,723 | 125.12 | 21,875,937 | 90.49 | 7.73 | 43.43 | 100 |
| ST_10^8^ CFU/mL | 4,258,020 | 138.69 | 4,230,865 | 139.15 | 1,514,710 | 35.82 | 58.08 | 70.17 | 100 |
| NT_10^8^ CFU/mL | 26,284,764 | 121.26 | 25,854,259 | 122.29 | 25,082,878 | 97.09 | 0.78 | 3.42 | 91.2 |
| ST_10^7^ CFU/mL | 18,085,854 | 135.18 | 17,966,513 | 135.6 | 14,897,757 | 82.97 | 8.12 | 39.66 | 100 |
| NT_10^7^ CFU/mL | 18,422,080 | 136.19 | 18,267,137 | 136.79 | 17,717,800 | 97.05 | 0.00 | nd | nd |
| ST | 18,805,544 | 131.88 | 18,638,349 | 132.49 | 17,349,942 | 93.15 | 0.00 | na | na |
| NT | 18,023,214 | 140.19 | 17,937,521 | 140.51 | 17,643,778 | 98.41 | 0.00 | na | na |
| **average** | **16,440,544** | **134.16** | **16,270,568** | **134.72** |  |  |  | **48.44** | **98.24** |
| Abbreviation: na, not applicable; nd, not detected | | | | | | | | | |

**Table S3.** Absolute DNA concentration and Ct values as reflected in Figure 3.

|  |  | **DNeasy** | | **AllPrep** | |
| --- | --- | --- | --- | --- | --- |
| Concentration *M. abscessus* (CFU/mL) | Condition | ng/µL | Ct value 16S rRNA gene | ng/µL | Ct value 16S rRNA gene |
| 1.21x10^9^ | ST | 2.81 | 14.28 | 2.24 | 14.41 |
|  | ST | 2.47 | 14.61 | 1.77 | 14.31 |
|  | ST | 3.56 | 13.81 | 1.64 | 14.79 |
|  | NT | 3.50 | 13.48 | 3.04 | 14.40 |
|  | NT | 2.50 | 14.41 | 2.73 | 14.36 |
|  | NT | 3.99 | 13.32 | 2.58 | 14.39 |
| 1.21x10^8^ | ST | 0.208 | 18.17 | 0.197 | 18.68 |
|  | ST | 0.146 | 18.45 | 0.137 | 18.84 |
|  | ST | 0.251 | 17.74 | 0.155 | 18.87 |
|  | NT | 0.235 | 17.69 | 0.550 | 18.20 |
|  | NT | 0.0924 | 19.90 | 0.659 | 20.15 |
|  | NT | 0.303 | 17.54 | 0.687 | 20.45 |

**Figure S1.** Fragment length of DNA extracted from samples that underwent the ST and NT. The x-axis represents the length in bp, and the y-axis represents the sample intensity (FU). Note that the y-axes differ in scale. **a**) ST, artificial sputum spiked with undiluted bacterial suspension; DNA extracted with DNeasy; highest peak at 13,679 bp. **b**) NT, artificial sputum spiked with undiluted bacterial suspension; DNA extracted with DNeasy; highest peak at 14,144 bp. **c**) ST, artificial sputum spiked with undiluted bacterial suspension; DNA extracted with AllPrep; highest peak at 17,460 bp. **d**) NT, artificial sputum spiked with undiluted bacterial suspension; DNA extracted with AllPrep; highest peak at 17,745 bp.


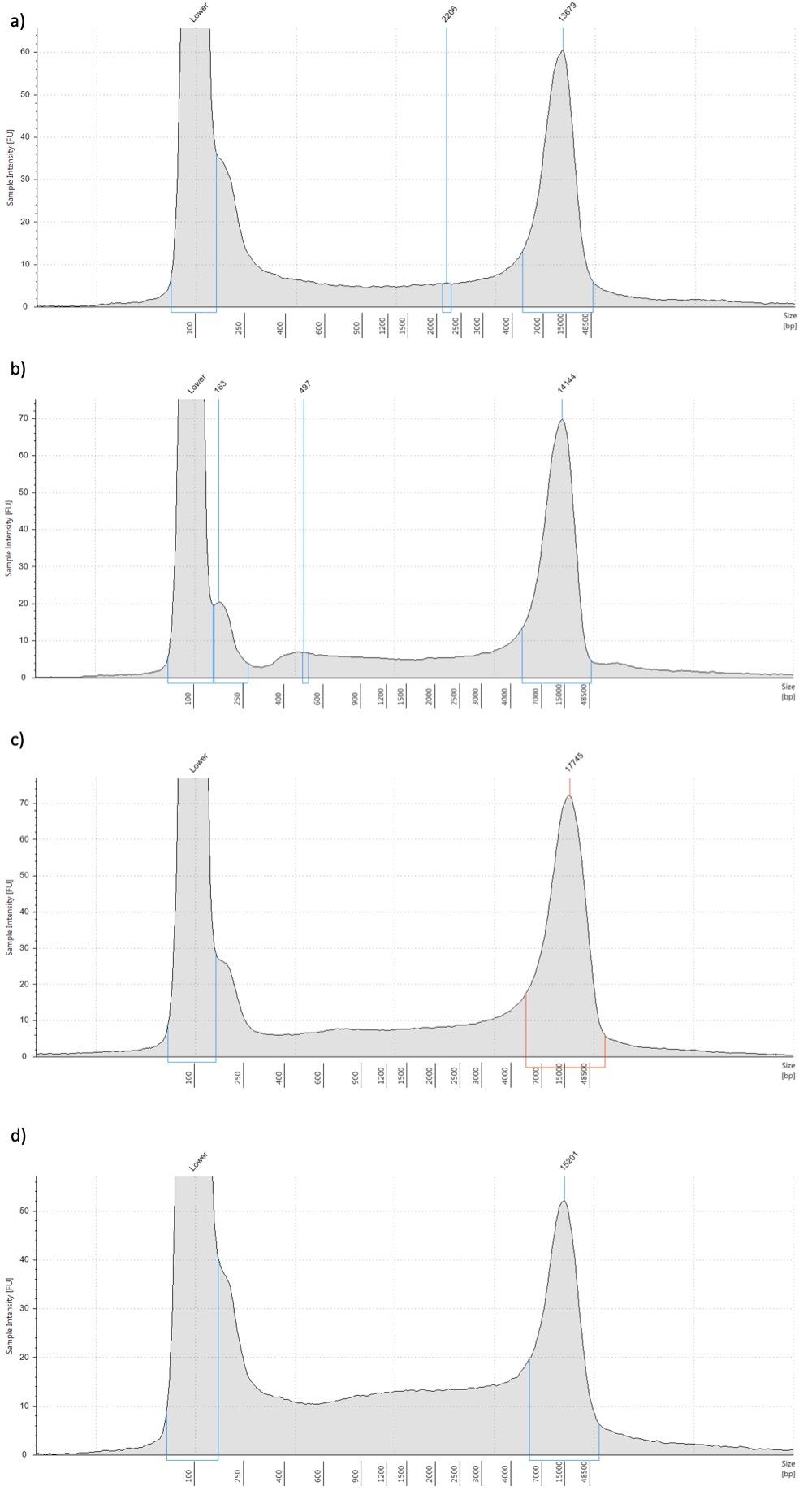


**Figure S2.** Fragment length of DNA extracted from samples that underwent the ST and NT. The x-axis represents the length in bp, and the y-axis represents the sample intensity (FU). Note that the y-axes differ in scale. **a**) ST patient sputum spiked with undiluted bacterial suspension; DNA extracted with AllPrep; highest peak at 15,201 bp. **b**) NT patient sputum spiked with undiluted bacterial suspension; DNA extracted with AllPrep; highest peaks between 385 and 778 bp.

**
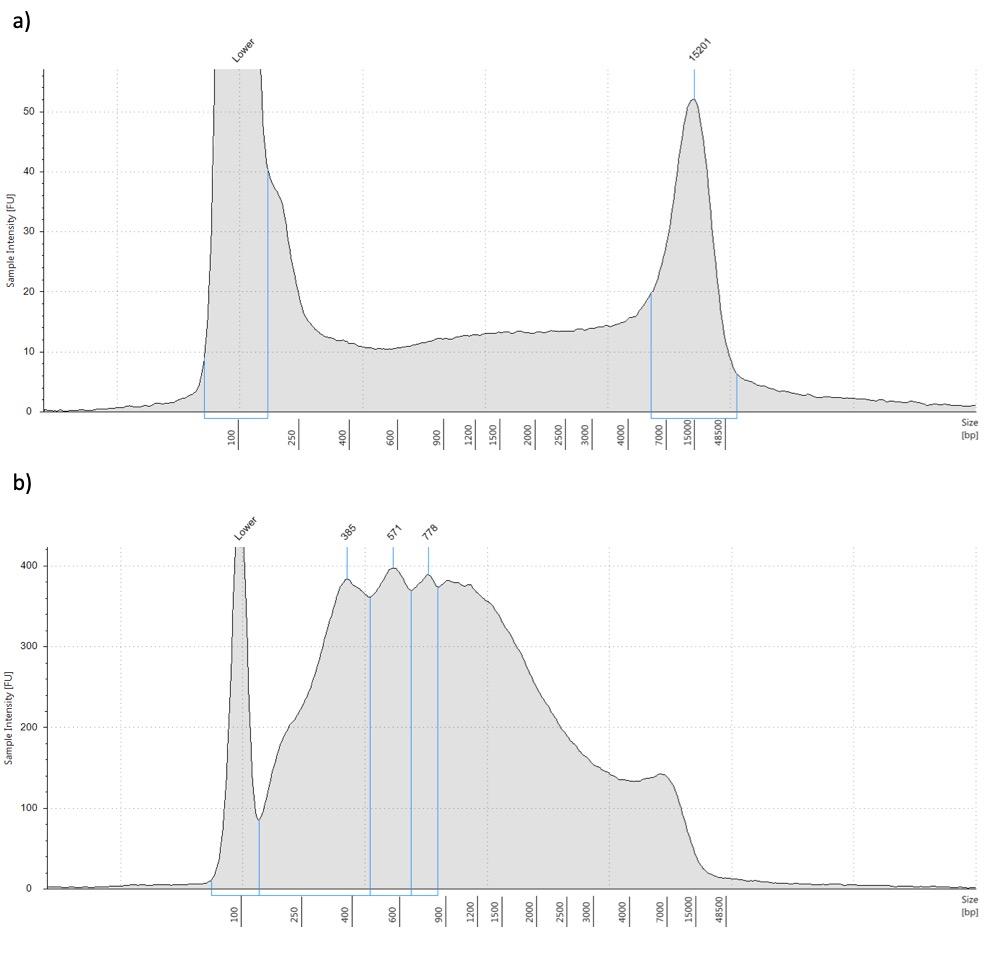
**

**Figure S3.** Effect of saponin treatment (ST) on virome composition of the patient sputum spiked with *M. abscessus*. Non-spiked sputa and sputa spiked with different concentrations of *M. abscessus* (high: 1.21x10^9^ CFU/mL, med: 1.21x10^8^ CFU/mL, low: 1.21x10^7^ CFU/mL). Subsequently, sputa were incubated (ST) or not (NT) with saponin followed by DNAse treatment.


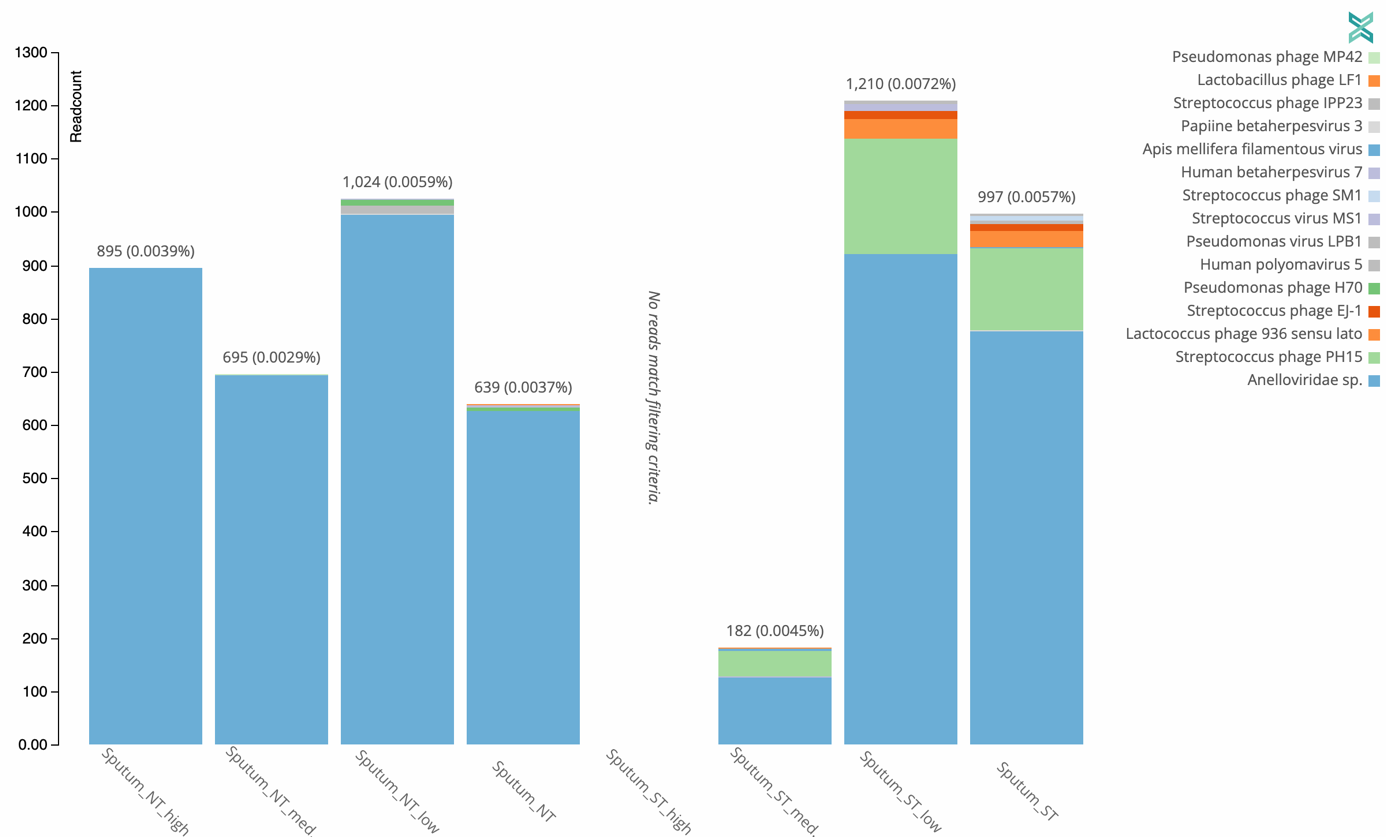

Supplement: Supplementary file 1 [file Data_Sheet_1.docx]
